# Falls and Falls Prevention in Singapore: Policy Insights From a Dynamic Simulation Model

**DOI:** 10.1093/geroni/igaf122.984

**Published:** 2025-12-31

**Authors:** Vanessa Koh, John Ansah, Angelique Chan, David Matchar

**Affiliations:** Duke-NUS Medical School, Singapore, Singapore; Case Western Reserve University, Cleveland, Ohio, United States; Duke-NUS Medical School, Singapore, Singapore; Duke-NUS Medical School Programme in Health Services & Systems Research, Singapore, Singapore

## Abstract

Falls among older adults have complex etiologies, requiring a multi-system approach for effective prevention. In Singapore, 85% of geriatric trauma cases presented in the emergency department result from falls. While multi-component falls prevention programs demonstrate evidence in reducing falls, their implementation remains challenging. Currently, Singapore lacks a national strategy to tackle falls, hence this study examines how such a strategy should be implemented to significantly avert falls. A Systems Dynamics model was developed to simulate the current reality of falls among older adults in Singapore. The model represents the resident population, patient-care continuum of falls prevention and capacity for screening and prevention services. Through different policy experiments, we aim to provide policy insights into the bottlenecks for intervention, and a combination of strategies that should be prioritized to optimize the impact of falls prevention programs. The model was simulated from 2010 to 2040, to project the impact of policies across a 30-year period. Five key bottlenecks in the current environment for falls have been identified. Policy experiments simulated changes in patient uptake, program quality, and capacity of services to alleviate these barriers. Findings suggest that an appropriate combination of investing resources for capacity expansion of screening and primary prevention programs, coupled with increased older adult engagement, improves the utilization of falls prevention services and health outcomes within 5-10 years after policy implementation. These findings demonstrate the need for a coordinated, resource-optimized approach to falls prevention, providing critical insights to inform policymakers in designing a national falls prevention strategy.

